# Mental Health Without Well-being

**DOI:** 10.1093/jmp/jhab032

**Published:** 2021-10-20

**Authors:** Sam Wren-Lewis, Anna Alexandrova

**Affiliations:** 1 University of Nottingham, Nottingham, England; 2 University of Cambridge, Cambridge, UK

**Keywords:** definition of health, happiness, medicalization, mental health, psychological flexibility, well-being

## Abstract

What is it to be mentally healthy? In the ongoing movement to promote mental health, to reduce stigma, and to establish parity between mental and physical health, there is a clear enthusiasm about this concept and a recognition of its value in human life. However, it is often unclear what mental health means in all these efforts and whether there is a single concept underlying them. Sometimes, the initiatives for the sake of mental health are aimed just at reducing mental illness, thus implicitly identifying mental health with the absence of diagnosable psychiatric disease. More ambitiously, there are high-profile proposals to adopt a positive definition, identifying mental health with psychic or even overall well-being. We argue against both: a definition of mental health as mere absence of mental illness is too thin, too undemanding, and too closely linked to psychiatric value judgments, while the definition in terms of well-being is too demanding and potentially oppressive. As a compromise, we sketch out a middle position. On this view, mental health is a primary good, that is, the psychological preconditions of pursuing any conception of the good life, including well-being, without being identical to well-being.

## I. INTRODUCTION—A VALUE IN SEARCH OF A DEFINITION

What is it to be mentally healthy? In the ongoing movement to promote mental health at work and in schools, to reduce stigma and to establish parity between mental and physical health, there is a clear enthusiasm about the concept of mental health and a recognition of it as a central value in human life. However, it is much less clear what mental health means in all these efforts and whether there even is a single concept underlying them. Sometimes the initiatives for the sake of mental health are aimed just at reducing mental illness, thus implicitly identifying mental health with the absence of diagnosable psychiatric disease. More ambitiously, there are also prominent initiatives in public health and policy that adopt positive definitions identifying mental health with psychic or even overall well-being. Nevertheless, those looking for an explicit agreed-upon definition will be disappointed.

In academic philosophy, and the philosophy of psychiatry in particular, discussions predominantly focus on the status of mental illness—whether it designates a natural kind or instead a social kind, whether classifications of disorders such as the Diagnostic Statistical Manual (DSM-5) should be based on symptoms rather than on underlying causes, and whether pharmaceutically driven interventions have too high of a profile by comparison to talk therapy.^1^ These are important and fascinating issues, and mental health connects with them all. However, in the case of mental disorders, these discussions are based on psychiatric practice: for example, the depressive disorders can be defined by persistent and debilitating sadness and listlessness. Notably, there is no corresponding uncontroversial source for the content of claims about mental health—there is no DSM for health. This is why what it means to be healthy is not at the forefront of today’s philosophy of psychiatry.^2^

At the same time, the definition of mental health is of deep philosophical as well as practical importance. On the practical side, if mental health is a state of well-being, efforts to promote it will likely adopt different outcome measures and methods of management, with different levels of trust invested in traditional psychiatric approaches. On the philosophical side, it is critical to start a conversation that, while implicit throughout the history of philosophy, has yet to happen explicitly: is mental health identical to well-being, is it one of its constituents, or a precondition for it?

The goal of our present discussion is to map out the goalposts of such a conversation. We do so by adopting something of a Goldilocks strategy. We identify a definition of mental health that is clearly too thin and undemanding—mental health as absence of mental illness—and a definition that is too ambitious and too demanding—mental health as the state of general well-being across all aspects of social and personal life. There are compelling reasons to reject both: the first one ties mental health too closely to the controversial concepts and methods of psychiatry, while the second one threatens to set up an impossible ideal, to medicalize unhappiness, and to dress up controversial philosophical judgments about the good life in the pretense of scientific objectivity.

It is this latter reason that led philosopher Simon Keller to pronounce the positive ideal of mental health to be dangerous—he points out that the nature of well-being is a matter of ethical and personal reflection and as such admits of great diversity. Once well-being becomes a condition of mental health, this invites scientific and medical recipes for good life, which are at best invalid and at worst oppressive.^3^ We readily agree that this is the danger of defining mental health in terms of well-being, but it does not have to be so. We sketch out a more attractive middle position. On this view, mental health is a primary good, that is, the psychological preconditions of pursuing any conception of well-being. This definition, we argue, is independently plausible and not dangerous.

## II. THE NEGATIVE DEFINITION

It makes sense to start by examining the minimalist position. It is based on a deeply plausible, almost tautological, observation: it is the business of medicine to treat disease and when this is successful, we have health. Since psychiatry is the branch of medicine dedicated to treating mental illness, psychiatry succeeds in its goal of restoring patients to mental health when it treats their psychiatric illnesses, where these illnesses are defined relative to standard classifications such as the DSM-5.

This definition is often endorsed implicitly rather than explicitly. For example, the current efforts to create parity between mental and physical health typically aim at improving access to treatment, while the campaigns promoting “mental health awareness” often mean little more than removing the stigma around mental illness and starting conversations about its causes such as stress.^4^

Underlying the intuitions that mental health is largely about tackling mental illness is an explicit philosophical position; it exists thanks to Christopher Boorse’s writings from 1970s onwards. Boorse is well known for his so-called biostatistical theory, which identifies health with normal species functioning. One statement of this view is as follows:

An organism is healthy at any moment in proportion as it is not diseased; and a disease is a type of internal state of the organism which:

(i) Interferes with the performance of some natural function—i.e., some species-typical contribution to survival and reproduction—characteristic of the organism’s age; and

(ii) Is not simply in the nature of the species, i.e., is either atypical of the species or, if typical, mainly due to environmental causes. (Boorse, 1976, 62–3)

The “biostatistical” label picks out the simultaneous importance to this view of (a) evolution by natural selection as a way of identifying functions and (b) of typicality of a given condition relative to others in this population. Boorse’s main goal was and remains a purely descriptive, value-free conception of health. It is a theoretical concept in physiology rather than in clinical medicine and as such it is sharply separate from normative questions of how individuals ought to function and to which conditions of their bodies and minds medicine should attend (Boorse, 1977, 1997). For these latter normative questions, Boorse proposes the notion of illness, that is, those diseases that are concerning enough to warrant medical treatment (Boorse, 1975). Boorse emphasizes that it is a further question—a question for ethics and political philosophy—whether a disease should be treated by doctors, covered by health care, and be the subject of clinical research. He might say the same for mental illness. Whether a given mental pathology should be considered a mental illness is ultimately an evaluative question. For instance, homosexuality, which at the time that Boorse was writing was being removed from DSM-III, may well be a disease on a value-free definition because it interferes with the function of reproduction, but it need not be an illness. In contrast, feminism and opposition to the Vietnam War—also controversies in the 1970s—have no basis for being considered psychiatric diseases, no matter how abnormal they might have seemed relative to prevailing social norms. Boorse positioned his theory against “normativism,” which attempted to ground health in some normative ideal and considered the value-freedom of his view to be its major advantage. In a less-known paper, “What a Theory of Mental Health Should Be,” Boorse extended the biostatistical theory to the case of mental health (Boorse, 1976). He argued that the extension is not problematic in principle. So long as we are able to formulate mental as opposed to physical functions of the organism, the atypical disfunction of one or more of these processes counts as a mental disorder. What might those functions be? Boorse recognized that it is easier to identify functions that underwrite physical health—heart to pump, sweat to maintain body temperature, liver to filter blood, and so on—than it is to pin down the mental functions that enable reproduction and survival. But he ventured that it is possible and in fact rehearsed one such theory—psychoanalysis. He wrote:

Formally speaking, psychoanalytic theory is the best account of mental health we have. It closely follows the physiological model by positing three mental substructures, the id, ego, and superego, and assigning fixed functions to each . . . . It would not be difficult to construe psychoanalytic theory as a set of theses about biological functions of the mind. On this view the id might emerge as a reservoir of motivation, the ego as an instrument of rational integration and cognitive competence, and the superego as a device of socialization. One could then give a straightforward argument that neurosis is disease by appealing to its disturbance of the integrative and motivational functions of the ego and the id. (Boorse, 1976, 78).

It is a notable historical irony that, just as psychoanalysis and all its attendant categories of trauma, repression, sublimation, and so on were getting purged from DSM-III,^5^ Boorse chose to ground his account of mental health, ostensibly scientific and objective, in the theory that was quickly losing ground in the psychiatric consensus. Now in his view, there were strong reasons for this choice. He was taking a stance against “normativists” who identified mental health with adjustment, normality, or well-being. The biostatistical theory was for him the only viable option unless one was willing to grant psychiatry the power to judge people.

For us, the question is not whether Boorse’s particular account of mental health is plausible—40 years later, the standing of psychoanalysis in psychiatry is even weaker than it was in the 1970s. Rather, our question is whether, despite Boorse’s conviction, there is any plausible formulation for a biostatistical theory of mental health.

## III. OBJECTIONS TO THE BIOSTATISTICAL THEORY

Boorse’s formulation of the fundamental contrast between normativism on the one hand and descriptivism (sometimes also known as naturalism) on the other has defined philosophical debate ever since. This discussion has predominantly focused on the definition of physical health. There are still defenders of naturalism (Hausman, 2011, 2015), but also of normativism (Cooper, 2002) and of a third option called hybridism (Wakefield, 1992). In addition to this, there are philosophers who reject the very possibility of ever settling a single precise definition of what might be a messy and disordered concept of disease (Worrall and Worrall, 2001; Ereshefsky, 2009). Those who criticize the biostatistical theory do so on the grounds that it lacks a defensible and value-free account of functions and of the right reference class against which to judge typicality. Critics have argued that it collapses under the weight of counterexamples; that is, cases where malfunctioning does not intuitively count as disease or good functioning that does intuitively count as such (Worrall and Worrall, 2001; Kingma, 2007, 2009).

Little of this large literature paid any attention to Boorse’s attempt to account for mental health. However, in our view the problems that philosophers have uncovered in the process of arguing with Boorse about physical health are severe enough to make it unlikely that a value-free articulation of mental functions is possible. Certainly, no one to our knowledge has tried to put forward an alternative to Boorse’s original psychoanalytic version of mental health; the attempts to reduce mental disorders to disfunction at the neurological level are, while ongoing, controversial (Kendler et al., 2011; Cooper, 2014b). While it is eminently plausible that some psychological capacities for judgment, valuation, and choice do make a clear contribution to survival and reproduction, the hard part is to articulate the overall set and to map this set on the existing categories in psychiatric classifications. Which of the evolved mental functions should operate, at what level and in which environment for a person to count as mentally healthy? The question seems intractable as our contemporary environment differs so much from our ancestral one; it is hard to know how we would even begin to find out whether, say, anxiety—clearly an evolved response to dangerous situations—will emerge as a disorder in this picture or not. This is one reason to reject Boorse’s naturalism.

Another reason is his implausible insistence on value-freedom. While for Boorse value-freedom was an essential guard against politicization of medicine in general and psychiatry in particular, his solution of distinguishing between disease (value-free) and illness (value-laden) only delays the necessity of making value judgments to another stage, without eliminating this necessity. Today’s demands on the concept of mental health are very much action-guiding. Scientists who deploy definitions of mental health deploy them for the most part not as Boorsean “theoretical concepts” relevant only to dispassionate and pure scientific research. Instead, their goal is deeply practical—how and what to diagnose, how and what to manage, how and what to prevent. For these purposes, Boorse’s definition of mental health does not provide the required normative guidance. This is unsurprising, given that Boorse does not think such guidance should be expected from the concept of health, so from his point of view this is not a weakness. Nevertheless, this lack does show the limited usefulness of his account.

Furthermore, it is far from obvious that the value-freedom of his account prevents politicization of psychiatry. Despite Boorse’s valiant efforts to articulate a value-free definition of disease, the value-ladenness of the DSM categories is by now well documented.^6^ What counts as a “sufficient impairment” or a “clinically significant” symptom are all locutions that conceal value judgments about the acceptable range of suffering and of deviation from norm. So if health is defined as absence of mental illness, then this definition is not all neutral, not at all naturalistic, because it inherits all the value judgments that inform definitions of depression, anxiety, attention deficit hyperactivity disorder, and so on.

Finally, we should question the very desirability of even aiming at value-freedom. Politicization of mental health is only threatening if *any* importation of values undermines the work this concept can do. Now it is far from clear that, just because some normative judgments have led psychiatry astray, any one of them would. Arguably, the problem was not that these judgments are normative, but that they are oppressive, cruel, and stigmatizing.

So there are three reasons to be concerned with Boorse’s version of value-freedom: first, it makes the concept of mental health ineffective in practice, second, grounding health in absence of disease does not in fact secure value-freedom if the existing definitions of disease are value-laden, and third, value-ladenness may not be as harmful as Boorse says it is. Together with the fact that a plausible account of mental functions does not exist, these create compelling reasons to look for a different account of mental health.

Now to arrive at such an account we need to consider our central objection to the negative definition of health inherent in Boorse’s biostatistical theory, an objection that other critics have left entirely untouched, at least to our knowledge. That is, that there is more to mental health than the absence of mental illness. This issue—whether mental health is negative or positive—is entirely orthogonal to the previous controversy between naturalism and normativism. Boorse is a naturalist and a negativist, so to speak, but it is conceivable that naturalism can be paired up with a positive definition of health or that normativism be paired up with a negative one.

We have seen reasons to depart from naturalism about mental health, but why depart from a negative definition? Is health more than the “silence of the organs?”^7^ Boorse himself rejected the calls for a positive definition—they were prominent already in the 1970s—as entirely confused (Boorse, 1977, 568–70). The relevant medical concept is the negative one and the positive concept is mostly a rhetorical tool for enthusiasts of fitness, dieting, and other healthy living movements. Sure, prevention of disease is important, and these activities might be effective for that purpose, but nothing more than the standard negative definition is needed for that. Ideas that go under the label of positive health are only tools for reducing disease.

Those who insist on adding a positive dimension to absence of disease will have to deal with problems that to Boorse seemed unsurmountable. First, any positive characterization seems utopian because there is no natural limit to it in the way that there is with absence of disease. One can cure an illness but it is hard to see how one can achieve full happiness, or full fitness. Second, different positive dimensions of health can be incompatible with each other, for example, being a sprinter and being a marathon runner. A positive definition of health would have to find a way of balancing them. Finally, he sees ethical problems with mandating any particular ideal of positive health:

The trouble with calling physical or mental or moral excellence health is that it tends to unite under one term a value-neutral notion, freedom from disease, with the most controversial of all prescriptions—the recipe for an ideal human being. (Boorse, 1977, 572)

Boorse’s critique of grand and ambitious positive definitions of health is compelling; we will shortly add more reasons to be wary of them. Just as in the argument for value-freedom Boorse unfairly saddled normativists with failings of oppressive psychiatry, similarly here he is saddling the advocate of positive definition with an implausible version of it. If the positive definition is utopian or unattainable, it should indeed be rejected. Now must it be utopian and unattainable? Is there a version that is positive and yet realistic and attractive?

There is good reason to try to build one. The negative definition ties mental health to the immensely controversial categories of mental disorders of contemporary psychiatry and nothing else. We have already seen that value judgments are already part and parcel of the definition of health on the “absence of disease” model. If so, why not rethink them with positive value judgments? A positive definition of health can act as a check on the values that ground the negative one, and it can explain why absence of disease is a good state to aim at. This promise obviously depends on the availability of a defensible positive conception; in many ways, we have already jumped ahead of ourselves. What are these demanding positive conceptions of which Boorse is so critical, and what exactly is wrong with them?

## IV. THE DEMANDING POSITIVE DEFINITIONS

Perhaps the best-known definition of mental health is articulated by the World Health Organization:

Mental health is defined as a state of well-being in which every individual realizes his or her own potential, can cope with the normal stresses of life, can work productively and fruitfully, and is able to make a contribution to her or his community. (World Health Organization, 2004)

This statement harks back to WHO’s definition of health, which appeared in its inaugural 1948 Constitution: “Health is a state of complete physical, mental and social well-being and not merely the absence of disease or infirmity.” Mental health according to WHO is also a positive state: it is not enough to be free of depression, anxiety, or schizophrenia, or any other diagnosable psychiatric condition; one also needs to be well enough to thrive and flourish in one’s community.

This definition is remarkable in more than one way. First of all, it is incredibly demanding: it describes a life in which individuals realize their full potential, as well as work productively and contribute to their community. Not many people meet such high standards. It is not clear exactly how high of a standard the definition endorses, but read literally, the standard is very high. A lot of seemingly well-functioning people would plausibly fail this test. It is possible that many of us are indeed mentally unhealthy, but it is also possible that this definition sets the bar too high. Second, it is an explicitly objective definition in that it demands that individuals actually meet these standards, not just think they do. Finally, it demands functioning in harmony with distinctly modern norms—these norms set what counts as normal stress, productive work, and contribution to society. The ethos of this definition is that one should not be a burden to one’s community: that is, when bad things happen to persons, they should be able to bounce back, and in particular, they should be able to hold a job and do their bit. Depending on how the expression “normal stresses of life” is cashed out, the definition can be interpreted as demanding that people do or do not expect to be rescued from unemployment or debilitating disease; in this sense, the definition implicitly refers to the modern welfare state. This is unsurprising: the 1948 definition of health was born just after World War II when the public health services were being institutionalized at the level of nation states and integrated into governance, while the more recent definition of mental health was put forward with heavy participation of economists from the World Bank.^8^

The WHO definition is not unique in being demanding. In 1958, following a wide-ranging review of literature on mental health and illness, British-Austrian sociologist Marie Jahoda articulated an influential and perhaps an even more demanding ideal, encompassing six criteria:

1. Self-acceptance and self-confidence
2. Self-actualization
3. Integration of different psychological functions
4. Autonomy
5. Accurate perception of reality
6. Environmental mastery (Jahoda, 1958)

Unlike WHO, Jahoda does not mention work and community contribution, but she adds the truth and accuracy requirement. Her goals are distinctly humanistic. She wishes to move beyond the emphasis on “adjustment” as the key aspect of mental health in the tradition of Karl Menninger (Menninger, 1930). But in rejecting this identification of mental health with social acceptability, she introduces an individualistic ideal based on the goals of excellence, competence, and autonomy.

Today, positive definitions of mental health have taken a distinct turn inspired by positive psychology. They have grounded mental health in well-being, flourishing, and happiness. Scientists in this tradition refer to “flourishing,” “mental wealth of nations,” and a number of other positive concepts that describe individuals’ psychological resources.^9^ These definitions are weaker in some sense but stronger in another. They are weaker in that the well-being in question is subjective rather than objective, but stronger in articulating specific positive emotions such as happiness, life satisfaction, or sense of flourishing.

What might be wrong with this?

## V. AGAINST DEMANDING DEFINITIONS

The proposals we mentioned above differ from each other in subtle ways and should ideally be examined individually. Now for present purposes, it is sufficient to identify a few concerns. Not all undermine each of the demanding options to the same extent; together, they nicely illustrate the problem.

The first problem is medicalization of unhappiness, idleness, dependence, and so on. Setting a given level of a positive good as a condition of mental health turns this good into a medical category when it was not previously so. Medicalization has a complex history where progress and humanism are intertwined with use of medicine for control and oppression (Conrad, 2007). Treating substance addiction as a medical problem rather than a moral failing is a positive illustration, but it is far less clear whether the failure of a child to conform to the modern classroom is an illness. The latter worries motivate critics of the modern scale of medicalization who point out that extending the domain of psychiatry actually undermines its epistemic and moral legitimacy (Charland, 2013).

It is not clear that any of the advocates of the demanding positive definitions are aware of these dangers. Take public health—clearly an important business of local and national governments. Political theorists of all stripes agree that the pursuit of this business carries dangers of oppression, such as when a government-backed ideal, in this case an ideal of mental health, is imposed on individuals in violation of their autonomy and self-determination. In the liberal tradition, such an imposition is a violation of the much-valued condition of neutrality, but in other intellectual traditions, such as the critical theory, medicalization is one of the instruments of maintenance and reproduction of an existing social order.

If it were possible to formulate a nonoppressive ideal of mental health (which we believe is possible), the intent to identify it with well-being or the good life raises difficult epistemic and conceptual issues regarding which notion of well-being to use and which elements to incorporate into “overall well-being.” Philosophers and social scientists typically treat well-being as an inclusive good, encompassing not just happiness, but also goods such as friendship, love, achievement, sense of purpose, and so on. How to weigh these goods in relation to each other is surely a deeply personal matter, and it is not clear whether a defensible recipe exists at the population level (Hausman, 2015; Alexandrova, 2017). It is precisely this problem—fitting all that well-being encompasses in one valid and practical measurement procedure—that motivates recent criticisms of well-being interventions in public health, such as the one articulated by England’s Chief Medical Officer:

After reviewing the evidence I conclude that well-being does not have a sufficiently robust evidence base commensurate with the level of attention and funding it currently receives in public mental health at national and local government level. Well-being, as a field within mental health, has not evidenced an acceptable definition or set of metrics. It is unclear how concepts and measures that do exist relate to populations with mental illness. (Davies, 2014, 15)

This harsh verdict is not uncontested (Huppert and Ruggieri, 2018) and the measurement of well-being remains a lively project in the social and medical sciences. However, whether measures of mental health should be identical to measures of well-being is and should be controversial.

Where does this leave us? The negative conception is not positive enough, while the existing positive ones are too positive, so to speak. Is there a happy medium? We submit there is, namely, a relatively value-neutral definition of mental health that avoids the problems associated with defining mental health in terms of well-being. To see where this more ecumenical conception lies, it is useful to analyze how it differs from common definitions of mental health. For instance, the following definition by the Public Health Agency of Canada is a fairly typical instance of how mental health is conceptualized by health practitioners and within public policy:

The capacities of each and all of us to feel, think, and act in ways that enhance our ability to enjoy life and deal with the challenges we face. It is a positive sense of emotional and spiritual well-being that respects the importance of culture, equity, social justice, interconnections, and personal dignity.^10^

How does our definition of mental health differ from this kind of conception? We wish the authors had stopped after the promising first sentence and skipped the overly grand second sentence altogether. What if we identified mental health not with well-being, but rather with those psychological capacities that, if developed and maintained, enable individuals to pursue any conception of the good life or well-being, whatever conception of it they adopt? In the following section, we flesh out the idea that mental health is a precondition of well-being, without being identical to well-being.

## VI. MENTAL HEALTH AS A PSYCHOLOGICAL PRIMARY GOOD

Let us begin with that first sentence from the Public Health Agency of Canada definition, which takes mental health to consist in “the capacities of each and all of us to feel, think, and act in ways that enhance our ability to enjoy life and deal with the challenges we face.” There are four notable features of this one-sentence definition, which we will briefly consider in turn before formulating our more general definition of mental health.

First, mental health concerns our *capacities* to feel, think, and act in certain ways that enhance our *ability* to attain certain states of affairs. It is not defined by how we actually feel, think, and act or by what we actually attain. Already these qualifications limit the scope of mental health in a liberal direction. This has much in common with the “capabilities approach” toward human development and justice (Nussbaum, 2000; Sen, 1999). According to the capability approach, the good life is not about having a set of goods, but rather about having a set of valuable capabilities. Valuable capabilities include freedoms to undertake valuable activities (e.g., holding a decent job, being able to engage in politics, being able to spend time in unspoilt nature) or freedoms to enjoy certain states of being (e.g., being healthy, being respected for one’s religious affiliation or sexual orientation, being able to live in a loving family and a supportive social network) (Robeyns, 2017). The advantage of focusing on capabilities—the abilities, skills, and opportunities—rather than actual states of doing and being is that people remain free to decide whether, and how best, to use them. Out of all capabilities our definition of mental health will be grounded in the psychological ones to feel and to think and we shall reserve for them the term “capacities.”

This focus on human capacities also coheres with George Graham’s definition of mental disorder (Graham, 2013). Graham defines mental disorder as the inability to develop and exercise psychological capacities for “flourishing,” which he further defines as the capacities we are bound to need, no matter what life we decide to pursue. In defining mental disorder in this way, Graham is drawing explicitly on John Rawls’ notion of *primary goods*—all-purpose goods that people need, whatever their plans. According to Rawls, liberal states should provide citizens with the necessary means to pursue their own conceptions of the good. As long as the pursuit of one’s own conception of the good does not result in harm to others, it is not the business of the state to deem whether or not one’s own conception of the good is worthwhile. Although Rawls was concerned with the material and social basis for primary goods, his theory can be extended to the psychological conditions required for people to pursue their own conceptions of the good life. We believe this way of defining mental health is useful and avoids the problems associated with definitions that ground mental health in the notion of well-being.

The second notable feature of the above one-sentence definition of mental health is that it is minimal. Instead of a long list of capacities, it consists in two: namely, the ability to enjoy life and the ability to deal with challenges we face. These capacities are broad and can be specified in a number of ways. For reasons of liberal neutrality, we consider this to be a virtue of a definition of mental health, rather than a vice. Recall that the Public Health Agency of Canada definition goes on to specify how these capacities are realized, stating that mental health is “a positive sense of emotional and spiritual well-being that respects the importance of culture, equity, social justice, interconnections, and personal dignity.” We can see how outlining the breadth of mental health in this way can be useful—showing how it may be impacted by a wide range of conditions, such as culture, equity, social justice, interconnections, and personal dignity. Although this may be true for some individuals, it may not be the case for others. The conditions that are actually required for people to enjoy life and deal with challenges they face is largely an empirical question and is partly determined by differences in personality, age, context, and culture.

Third, the above definition concerns capacities to *feel, think, and act* in certain ways. This emphasis on *psychological* capacities—feeling, thinking, and acting—distinguishes it from definitions of physical health. Definitions of physical health (positively construed) are concerned with the *physiological* capacities that enhance our ability to enjoy life and deal with the challenges we face. This does not mean to say there are not significant areas of crossover between the two, for example, diet, exercise, social connection. However, we believe the above distinction accounts for the main differences between physical and mental health treatment.

Fourth, it is worth noting the two broad capacities that mental health consists in according to the above definition: (a) to *enjoy life* and (b) to *deal with the challenges we face*. These broad capacities get to the heart of what it means to be mentally healthy. However, we believe they can be even more broadly construed to qualify as a psychological primary good. First, we can re-construe the ability to enjoy life as the ability to *value life*—to see life as valuable or worth living. Not everyone needs to enjoy life to pursue their own conception of the good life. However, the ability to value life is a necessary precondition for well-being. We can reconstrue the second capacity in a similar way. The ability to deal with the challenges we face is an important component of the wider ability to *engage in life—*to pursue the things in life that seem valuable or worthwhile, despite difficulties, challenges, setbacks, failures, losses, and so on.

Although these capacities come in degrees, they nonetheless can fall above or below certain thresholds. For instance, one symptom of depression is that patients are unable to see value in themselves, others, or their environment. Similarly, patients with general anxiety disorder, due to excessive worry and anticipation of disaster, may be unable to cope with various demands and threats concerning the things they care about. In both cases, patients can be viewed as slipping below a certain threshold of being able to value or engage in life.

On the basis of these four points, we can offer the following one-sentence definition of mental health as, “the capacities of each and all of us to feel, think, and act in ways that enable us to value and engage in life.” In the next section, we outline what we take these psychological capacities for valuing and engagement to consist. Note that we only offer this outline as a way of clarifying the scope of this relatively neutral definition of mental health. It is a starting point for a full, empirically informed account, which is beyond the scope of this article.

## VII. TOWARD A BROAD DEFINITION OF MENTAL HEALTH

We maintain that mental health can be defined as “the capacities of each and all of us to feel, think, and act in ways that enable us to value and engage in life.” In this section, we expand on what the two main features of this definition—valuing life and engaging in life—consist.

Consider valuing life, first. We take this to consist in capacities to care about certain states of affairs—features of ourselves, others, and our environment. When one values something, one is positively disposed toward it (in a psychological sense) in various ways (Tiberius, 2018). For example, when one loves someone, one may feel a sense of warmth and connection around them, a mild form of separation distress when they leave, be motivated to care for them and look out for their well-being, and so on. This is different to merely seeing something as good or valuable in some way. Valuing, in addition, involves being disposed or committed to seeing something as valuable. This may not always take the form of an explicit value judgment toward that thing (i.e., “I value *X*” or “I believe *X* is worthwhile”) but nonetheless influences how one consistently feels, thinks, and acts in relation to it over time.^11^

Our capacities to value certain states of affairs may seem relatively straightforward or foundational, but they still need to be developed, and may remain fragile throughout our lives. Psychopathy, for instance, can be viewed partly as an inability to develop certain valuing capacities, in particular, the ability to value the well-being of others. Alternatively, individuals may lose their valuing capacities, as can be the case with depression, or during extreme cases of grief. To be able to appreciate life is foundational to our sense of agency, but it is far from straightforward. Valuing certain states of affairs—features of ourselves, others, and our environment—is a complex endeavor, which may, among other things, require the presence of basic goods, such as pleasure, loving relationships, moral worth, and good effects, and the absence of their opposites (Smuts, 2017).

Let us now turn to the second part of our proposed definition of mental health, namely, engaging in life. This includes the ability to “deal with challenges we face,” as emphasized in the Public Health Agency of Canada definition above. Again, this is just a sketch, but we take engaging in life to at least partly consist in the capacities that make up *psychological flexibility*.^12^ Psychological flexibility is “the ability to change or persist in behavior when doing so serves valued ends” (Hayes et al., 2011). In the pursuit of valued ends, we often come across difficulties, challenges, setbacks, failures, and losses. In response, being psychologically flexible enables us to shift our perspective, successfully adapt, and learn from our situation. This may result in either changing or persisting behavior, depending on what is learned. For instance, the practice of mindfulness often involves subjects simply paying attention to their sensations, thoughts, and feelings—neither ignoring them nor seeing them as the full picture. This can help subjects act on the information provided by those sensations without engaging in habitual patterns of meaning and behavior.^13^

As with our capacities to value life, our capacities for engaging in life also need to be developed, and may remain fragile in the response to various difficulties, setbacks, and adversities. Mental disorders such as depression and general anxiety disorders often involve individuals “getting stuck” in certain ways of thinking, feeling, and acting. We may often be able to pursue our values through the formation of appropriate goals and plans. However, when things inevitably do not go to plan, we may be more or less able to treat our situation in a psychologically flexible manner. Psychological flexibility involves being able to see such situations as providing us with worthwhile—if not predominantly negative—information. We may use this information to form new goals and plans, and different sets of skills, habits, and behaviors. These abilities to understand and act within any given situation, or within any area of life, are foundational to a person’s mental health.

In summary, our definition of mental health builds on existing positive definitions of mental health, while stripping them down to their foundations. We focus on people’s basic abilities to form values and pursue them in the face of life’s difficulties and challenges. In short, we suggest that mental health consists in people’s basic psychological capacities to value and engage in life. These capacities are positive psychological features, not mere absence of mental illness, but they are not sufficient for well-being. The definition thus offers more than the negative one and less than the utopian ones.

## VIII. OBJECTIONS AND REPLIES

In this section, we consider some objections. According to our definition, mental health concerns the capacities of each and all of us to feel, think, and act in ways that enable us to value and engage in life. One might object that both of these two main features of the definition—valuing life and engaging in life—are problematic notions.

Consider, first, the notion of valuing life. One might object that our definition of valuing life does not consist in capacities for valuing things that are actually valuable. For example, according to our definition, someone might qualify as being mentally healthy, even if they value things that do not make them better off, such as fame and fortune. This is problematic insofar as mental health is typically viewed as something of value, either in an intrinsic or instrumental sense (Raibley, 2013). Why should we care about people’s mental health if it consists in people valuing things that do not contribute toward their well-being?

In response, we believe that we should embrace the potential for mentally healthy individuals to develop values and conceptions of the good life that may not in fact be good for them. Thus, according to our definition, mental health is a *necessary but not sufficient* precondition for well-being. Well-being requires not merely the ability to value certain states of affairs, but rather the ability to value states of affairs that are good for us. Instead of viewing this as a weakness of our definition, we take it to be a strength. In contrast to the notion of well-being that is defined relative to some substantive good such as pleasure, success, or flourishing, the concept of mental health makes reference only to valuing and engagement; these capacities are neutral with respect to the precise conception of well-being that an individual may adopt. We hope, or rather it is an empirical bet we are making, that whether one is a hedonist, an Aristotelian, or a desire theorist, certain capacities are fundamental. That these capacities are as we outlined above, is an empirical matter we want science or medicine to answer.^14^ This is not to say that our definition is purely descriptive. What it takes to value and to engage may well require value judgments (for instance, about what counts as a normal setback vs. an insurmountable obstacle that people should not be expected to overcome), but these judgments are less contentious than judgments about the nature of well-being.

Thus, our definition of mental health steers clear of normative judgments about what is good for us and how we ought to live. Mental health consists in being able to value certain features of ourselves, others, and our environment, rather than certain features we *should value* according to any particular prudential, moral, or religious theory.

The second objection relates to the notion of engaging in life. The ability of individuals to effectively pursue the things they value requires a range of attentional, emotional, and cognitive capacities (Mele, 1997; Holton, 2009; Bratman, 2018). In contrast, our definition of mental health only consists in the broad capacities that make up psychological flexibility—the ability to change or persist in behavior when doing so serves valued ends. One might object that this definition is relatively uninformative.

In response, although it is tempting to expand this list of capacities to include other psychological capacities (in particular, those related to the formation and maintenance of social relationships), we want to resist this move. A more expansive list may include the kinds of psychological capacities outlined in Jahoda’s definition of mental health discussed above—things such as self-acceptance and self-confidence, self-actualization, autonomy, and environmental mastery (Jahoda, 1958). However, we have argued that these more demanding definitions of mental health are problematic in various ways. In order to remain as value-neutral as possible, our definition of mental health consists in broad psychological capacities which can then be further specified, on a case-by-case basis, in accordance with a particular individual’s values, and other, more general, factors such as their personality, age, context, and culture.

The result is a definition of mental health that views mental health as a necessary (but not sufficient) precondition for well-being. Moreover, it leaves considerable room for empirical work to flesh out what mental health consists in for different cultures, groups, personalities, ages, and so on, and clinical work to determine the specific capacities that affect the mental health of any given individual. Being able to value and engage with life is likely to consist in a complex range of attentional, emotional, and cognitive capacities that will vary considerably by context. Nonetheless, our definition of mental health provides a rough outline of the broad kinds of psychological capacities that matter and why.

## IX. LOOKING AHEAD

We have argued that there exists a happy medium between the insufficiently and the overly demanding definitions of mental health. Our definition has the following features:

1. It is positive rather than negative because in addition to the absence of disease, we specify a further requirement.
2. It is grounded in well-being without identifying mental health with well-being.
3. It is politically legitimate or at least has a better chance of being so than the more demanding definitions.

By way of conclusion, we wish to raise two issues we have not discussed in this article but that may well be affected by our proposal. First is the issue of measurement. It is a live controversy which scales, questionnaires, and indicators best capture mental health. One popular scale for measuring “mental well-being” is the Warwick and Edinburgh Mental Well-being Scale, which asks people to judge the extent to which they feel optimistic, effective, useful, relaxed, able to get along with other people, and to make up their mind about things (Stewart-Brown and Janmohamed, 2008). Although it has the word “well-being” in it, in fact it measures people’s own sense of their personal effectiveness for going through with basics of life. There is a difference between this sense of personal effectiveness on the one hand and subjective well-being on the other. Intuitively, the latter refers to our emotional well-being, our happiness, our fullness of heart, joy, and contentment. Feeling happy takes more than just patting yourself on the back for being effective. Quite possibly, this scale captures our definition reasonably well, but this is ultimately a question for a proper psychometric investigation.^15^

The second issue is which interventions support mental health on our definition. This is of course an empirical question. However, it seems likely that mental health as a precondition of well-being would need more than medication and talk therapy. The development and the maintenance of the relevant psychological skills call for interventions at social rather than individual levels, anticipating structural obstacles to exercise of agency such as gender norms, racial and class discrimination, and generally aim at empowerment, rather than only treatment and prevention.

## Footnotes

1. For DSM-5, see American Psychiatric Association (2013); for general philosophy of psychiatry, see Fulford et al. (2013); for the status of mental illness, see Murphy (2006); for the influence of social factors on psychiatric research, see Cooper (2014a); and for a philosophical overview of DSM-5, see Cooper (2014b).

2. We note in particular that the Stanford Encyclopedia article on Philosophy of Psychiatry does not cover this issue (Murphy, 2017).

3. See Keller (2017) for the original blogpost and Keller (2020) for the article that develops this idea.

4. For examples of this in the English context, see McShane (2013) and Mental Health Foundation (2021).

5. See Decker (2013) on the processes and motivations that led psychiatrists such as Robert Spitzer and his colleagues to remove all Freudian language from the standard classification of mental disorders.

6.
Jahoda (1958), Hacking (2007), Hawthorne (2013), Cooper (2014a, 2014b), among many others.

7. This is an expression due to Rene Leriche, later explored by George Canguilhem (1991).

8. For a history of public health, see Rosen (2015). We thank Mildred Cherfils for explaining to us the role of the World Bank and economic considerations in the 2016 WHO campaign “Out of the Shadows: Making Mental Health a Global Priority.”

9. See Keyes (2002), Beddington et al. (2008), Huppert and So (2013), and Stewart-Brown (2013).

10.
Public Health Agency of Canada (2014).

11. Sometimes we may consistently see something as valuable without valuing it. This may be a result of habit or compulsion, as is often the case with addictive behaviors. In such cases, individuals may not identify with their feelings toward an addictive substance or behavior, instead seeing them as alien and contrary to their sense of agency. They may feel shame toward such feelings, pride at overcoming them, and admiration toward others who manage to do so.

12. For a review, see Kashdan and Rottenberg (2010).

13. For related notions to psychological flexibility, see De Young (2015) on the personality meta-traits of Stability and Plasticity, and McGilchrist (2012) on the divided brain.

14. It is possible that empirical findings will reveal that there is no single set of psychological capacities that underlie all the plausible conceptions of well-being. This is a possibility that we should certainly allow for, but equally ours is a justifiable bet.

15.
Böhnke and Croudace (2016) raise significant worries about the validity of all the existing measures of positive mental health.
